# Minimizing Stochastic Complexity with Ridge Regression

**DOI:** 10.3390/e28070735

**Published:** 2026-06-30

**Authors:** Antony Mizzi, David M. Walker, Michael Small

**Affiliations:** Complex Systems Group, Department of Mathematics and Statistics, The University of Western Australia, Perth, WA 6009, Australia; david.walker@uwa.edu.au (D.M.W.); michael.small@uwa.edu.au (M.S.)

**Keywords:** model selection, coding theory and techniques, information theory, regularization

## Abstract

We derive a penalty strength criterion for ridge regression using stochastic complexity, which is a refined variant of the minimum description length principle. Since stochastic complexity does not typically account for the effect of regularization on complexity, despite its ability to simplify models, we are required to make a slight modification to the underlying coding scheme. Our scheme makes use of a weighted ensemble of regularized model fits rather than a mixture of maximum likelihood estimates. Under this modification, regularization is interpreted as reducing model complexity by constraining flexibility. In the case of ridge regression, the complexity penalty term that we derive can be expressed analytically as the log determinant of the residual operator. We demonstrate the effect of this complexity penalty by fitting a linear readout to a reservoir computer, and by performing benchmark testing on publicly available datasets.

## 1. Introduction

We approach the problem of selecting a regularization strength for ridge regression with the intent of producing the simplest generalized linear model (GLM). This complexity-based approach is particularly relevant since the popularity of GLMs stems, in part, from their simplicity as a model class [1,2,3,4,5]. They are often used to identify and characterize relationships from data in fields like medicine [6,7], dietary science [8], economics [9,10], and sociology [11,12]. Such applications lie in accordance with Occam’s razor principle, which tells us to seek simple explanations, or in this case, simple model classes.

In these endeavours, it is common to use some form of regularization in the fitting process, like an L2 ridge penalty. In the case of ridge regularization, the penalty term stabilizes the matrix inverse under multicollinearity of input variables [3,13,14] and improves generalization to new data by reducing model variance at the expense of increased model bias [15,16,17]. The application of regularization also further reduces the complexity of linear models. This makes it possible to choose the regularization strength that minimizes model complexity.

In this paper, we measure complexity in terms of stochastic complexity [18], which is a refined variant of the minimum description length principle [19] (MDL). The stochastic complexity of a model is directly related to its flexibility. When used for model selection, it suggests that we should hesitate to choose a model class that fits our dataset well if it also provides good fits to unrelated datasets using the same predictors. While regularization constrains flexibility and produces simpler models, this is not directly accounted for by stochastic complexity. As such, the purpose of this paper, as well as deriving a ridge strength criterion, is to demonstrate a method for determining the effect of regularization on the stochastic complexity of a model. Our method makes use of a novel interpretation of stochastic complexity’s underlying coding scheme in order to directly account for the constraint imposed by regularization on model flexibility.

Our work is organized as follows. In Section 2, we provide an overview of stochastic complexity, including an explanation of the typical underlying coding scheme. In Section 3, we discuss our method for modifying this scheme to account for regularization. In Section 4, we derive an analytical form for the stochastic complexity of a linear model fitted by ridge regression using our modified scheme. We also present our ridge penalty selection criterion. In Section 5, we compare our criterion with several other information-theoretic criteria. In particular, we note that our work is closely related to that of Dwivedi et al. [20], who recently used luckiness normalized maximum likelihood [21] (LNML) for ridge strength selection. Their approach produces a different selection criterion, which stems from a different interpretation of regularization’s effect on complexity. In Section 6, we discuss the effect of our modification on stochastic complexity’s underlying probability distribution. We then visualize this effect using a two-dimensional toy example. In Section 7, we use our criteria to train linear readouts with the example of reservoir computing. We also perform benchmark testing in Section 8 using more than one hundred publicly available datasets from the Penn Machine Learning Benchmark (PMLB) repository [22]. In Section 9, we conclude with a discussion and summary.

## 2. Background into Stochastic Complexity and the Minimum Description Length Principle

The premise behind MDL is that models compress information. This is done by making use of a parametric model class to derive a coding scheme for possible data sets. The shorter the encoding of the dataset we wish to model under the scheme we derive, the better the compression achieved by the model. For example, in classical two-part MDL, the scheme is to first encode our model parameters and then to encode the errors that the model makes using these parameters. The best models, according to two-part MDL, minimize the combined lengths of their error and parameter codes.

The coding scheme assumed by stochastic complexity is more abstract. No one set of model parameters is used. Instead, a probability $P_{0}^{*}\left(Y;M,X\right)$ is assigned to each potential dataset *Y* from the set of possibilities Y∈Y using a weighted ensemble of parameterizations from the same model class M:(X,θ)→Y. If we denote the weighting associated with each parameterization θ in the ensemble as π(θ), then the probability assigned by the resulting ensemble would be equivalent to the marginal probability under π as a prior

$$P_{0}^{*}\left(Y;M,X\right)=\sum_{\theta }P\left(Y|\theta ;M,X\right)\pi \left(\theta \right).$$

Importantly, the ensemble weightings are chosen deterministically based on the model class. This is what enables the resulting code to be deciphered and makes stochastic complexity a valid description length. Under the stochastic complexity scheme, ensemble weights π(θ) are chosen before the dataset *Y* is known in the way that minimizes regret for the worst possible scenario of *Y*. This regret $R_{0}\left(Y,P_{0}^{*}\right)$ is the difference between the length of code assigned to *Y* by the weighted ensemble (which we denote by $L_{\mathrm{sc}}\left(Y\right)$) and the shortest length that could be used to encode *Y* with any other ensemble. Under the lower bound for efficiency given by Shannon’s source coding theorem, these code lengths are negative log probabilities $L_{\mathrm{sc}}\left(Y;M,X\right)=-\log\left(P_{0}^{*}\left(Y\right)\right)$. Since the best ensemble for encoding any particular dataset *Y* is just its maximum likelihood estimate $\theta_{Y}^{*}$, the worst-case regret which we seek to minimize by choice of weightings is the following

(1) $$\begin{array}{cc} R_{0}^{\max}\left(Y,P_{0}^{*}\right) & :=\max_{Y\in Y}\left(R_{0}\left(Y,P_{0}^{*}\right)\right), \end{array}$$

(2) $$\begin{array}{cc} \mathrm{where}\;R_{0}\left(Y,P_{0}^{*}\right) & :=-\log\left(P_{0}^{*}\left(Y;M,X\right)\right)+\log\left(P\left(Y|\theta_{Y}^{*};M,X\right)\right). \end{array}$$

The weightings that minimize this worst-case regret are those that induce proportionality to the maximum likelihood estimate ($P_{0}^{*}\left(Y;M,X\right)=\frac{1}{C_{0}}P\left(Y|\theta_{Y}^{*};M,X\right)$). This means that we can work directly with $P_{0}^{*}$ without ever actually determining the optimal weightings. Under proportionality, regret is constant across all datasets $R_{0}\left(Y_{1},P_{0}^{*}\right)=\log\left(C_{0}\right)\;\forall Y\in Y$. The constant of proportionality $C_{0}$ must be the sum of all maximum likelihoods in order for it to induce a properly normalized distribution that integrates to one. For this reason, $P_{0}^{*}$ is often called the normalized maximum likelihood (NML) distribution. The probability of *Y* under $P_{0}^{*}$ is given as follows

(3) $$P_{0}^{*}\left(Y;M,X\right)=\frac{P(Y|\theta_{Y}^{*};M,X)}{\sum_{Y^{\prime }\in Y}P\left(Y^{\prime }|\theta_{Y^{\prime }}^{*};M,X\right)}.$$

When we work with continuous data, and therefore an infinite set of possible datasets Y, the ensemble weights π(θ) become difficult to determine. They have been shown [4,23], to approach the Jeffreys prior [24] for large datasets under certain conditions [25]. However, we are not required to determine the ensemble weights since proportionality still holds, and we can again work directly with $P_{0}^{*}$. In the case of continuous data, the stochastic complexity makes use of the integral version for $P_{0}^{*}$

(4) $$L_{\mathrm{SC}}\left(Y;M,X\right)=-\log\frac{P(Y|\theta_{Y}^{*};M,X)}{\int_{Y^{\prime }\in Y}P\left(Y^{\prime }|\theta_{Y^{\prime }}^{*};M,X\right)dY^{\prime }}.$$

Notice that stochastic complexity can be broken into two terms, the first being the model’s negative log likelihood. The other term measures the complexity of the parametric model class M

$$\mathrm{Penalty}=\log\left(\int_{Y^{\prime }\in Y}P\left(Y^{\prime }|\theta_{Y^{\prime }}^{*};M,X\right)dY^{\prime }\right),$$

and functions as the penalty term in model selection. This penalty directly measures flexibility and it lends stochastic complexity an important intuition beyond its meaning as a code length. It tells us not to trust models that fit the data well if they can also provide good fits to unrelated data using the same predictors.

## 3. The Effect of Regularization on Complexity

The role of model regularization on stochastic complexity can potentially be interpreted in different ways. Our interpretation is to directly consider the effect of regularization on the flexibility of the model class. This follows on from the intuitive meaning of stochastic complexity described in the previous section: that we should have more confidence in our regularized model if the regularization applied prevents the model from easily fitting unrelated datasets using the same predictors.

Rather than building a weighted ensemble of maximum likelihood estimates, we build a weighted ensemble of regularized fits. The weightings of this ensemble will depend on the regularization strength α. We select the weightings such that the probability assigned by the ensemble $P_{\alpha }^{*}\left(Y;M,X\right)$ minimizes a modified form of regret in the worst case scenario. This modified regret, which we now denote by $R_{\alpha }\left(Y,P_{\alpha }^{*}\right)$, because of its dependence on the regularization strength α, is still the length of code that could have been saved had the parameter fit for the dataset been known ahead of time. The only difference is that this parameter fit is now the set of regularized parameters and not the maximum likelihood estimate

$$R_{\alpha }^{\max}\left(Y,P_{\alpha }^{*}\right):=\max_{Y\in Y}\left\{-\log\left(P_{\alpha }^{*}\left(Y;M,X\right)\right)+\log\left(P\left(Y|\theta_{Y,\alpha }^{*};M,X\right)\right)\right\}.$$

Assuming that the encoder and decoder agree on the regularization strength ahead of time, they can calculate the ensemble probability $P_{\alpha }^{*}$ that minimizes this modified regret in the worst case choice of the data set Y∈Y. We demonstrate in Appendix A that the solution is to choose $P_{\alpha }^{*}$ proportional to the likelihood at the regularized parameter fit $P_{\alpha }^{*}\left(Y\right)\propto P\left(Y|\theta_{Y,\alpha }^{*}\right)$. The constant of proportionality is the normalization factor required to produce a distribution that integrates to unity. In the continuous case, the resulting NML distribution is defined as follows

(5) $$P_{\alpha }^{*}\left(Y;M,X\right)=\frac{P(Y|\theta_{Y,\alpha }^{*};M,X)}{\int_{Y^{\prime }\in Y}P\left(Y^{\prime }|\theta_{Y^{\prime },\alpha }^{*};M,X\right)dY^{\prime }},$$

while in the discrete case, the integral is replaced with a sum. Because the normalization factor ensures that $P_{\alpha }^{*}$ is a valid probability mass, it follows from Kraft’s inequality that a uniquely decodable scheme exists to describe the elements of Y with code word lengths between $-\log(P_{\alpha }^{*}\left(Y\right))$ and $-\log(P_{\alpha }^{*}\left(Y\right))+1$ nats (Ignoring fixed precision costs for continuous datasets). The scheme that achieves these code lengths is well defined for most model classes and types of regularization, which we discuss in Appendix B. The code lengths it assigns to datasets are given by the following

(6) $$L_{\mathrm{SC}}\left(Y;M,X,\alpha \right)=-\log\frac{P(Y|\theta_{Y,\alpha }^{*};M,X)}{\int_{Y^{\prime }\in Y}P\left(Y^{\prime }|\theta_{Y^{\prime },\alpha }^{*};M,X\right)dY^{\prime }}.$$

One benefit of the model selection criterion that comes from this coding scheme ($L_{\mathrm{SC}}\left(Y;M,X,\alpha \right)$) is its generalizability. The criterion derived in Equation (6) is not specific to any particular model class or type of regularization. Another benefit is the fairness of the comparison it draws between regularization techniques. One technique can be compared with another based purely on how much each restricts the flexibility of the model class. The caveat, which is not unique to our approach, is that to derive an analytical value for stochastic complexity, we still need an analytical form for the model likelihood when regularization is applied.

## 4. Deriving a Ridge Penalty to Minimize Stochastic Complexity

In the case of regression with a known state matrix *X*, the parametric model class M is the multivariate normal distribution $N(X\theta ,\epsilon^{2}I)$. This is the model class for which the regression solution $\theta =\theta_{Y}^{*}$ provides the maximum likelihood estimate. From a Bayesian perspective, the ridge penalty comes from maximizing the posterior with the added assumption of a normal prior over the parameters. However, it is not strictly necessary to assume a prior over the parameters in order to calculate stochastic complexity. We only need to know the probability of each dataset Y∈Y given the regularized parameter fit $\theta_{Y,\alpha }^{*}$. According to our model class, that probability is the following

$$P\left(Y|\theta_{Y,\alpha }^{*};M,X\right)={\left(2\pi \epsilon \right)}^{-n/2}\exp\left(-\frac{||Y-X\theta_{Y,\alpha }^{*}{\left|\right|}^{2}}{2\epsilon^{2}}\right).$$

Fortunately, an analytical form for this probability exists. First, we can express the ridge regression solution $\theta_{Y,\alpha }^{*}$ in terms of the penalty α, the state matrix *X* and the dataset *Y*. It is given by $\theta_{Y,\alpha }^{*}={\left(X^{T}X+\alpha I\right)}^{-1}X^{T}Y$. This means that an analytical form for the mean square error (MSE), which we denote by $\sigma_{Y,\alpha }^{2}$, also exists

$$\begin{array}{cc} \sigma_{Y,\alpha }^{2}=\frac{1}{n}Y^{T}Q_{\alpha }Y,\;\mathrm{where}\;Q_{\alpha } & ={\left(M_{\alpha }-I\right)}^{T}\left(M_{\alpha }-I\right)\\ \mathrm{and}\;M_{\alpha } & =X{\left(X^{T}X+\alpha I\right)}^{-1}X^{T}. \end{array}$$

In the expression above, *n* refers to the length of *Y*. We can use the matrix form for mean square error to rewrite the NML probability $P_{\alpha }^{*}\left(Y\right)$ in Equation (5) for the context of ridge regression

(7) $$P_{\alpha }^{*}\left(Y;M\right)=\frac{{\left(2\pi \epsilon \right)}^{-n/2}\exp\left(-\frac{Y^{T}Q_{\alpha }Y}{2\epsilon^{2}}\right)}{\int_{Y^{\prime }\in Y}{\left(2\pi \epsilon \right)}^{-n/2}\exp\left(-\frac{Y^{\prime }TQ_{\alpha }Y^{\prime }}{2\epsilon^{2}}\right)dY^{\prime }}.$$

The stochastic complexity then comes from taking the logarithm of this probability. The component corresponding to the complexity penalty is found by evaluating the integral in the denominator. For continuous datasets and linear models, it makes sense to take the space of possible datasets to be all real valued vectors of length *n* and set Y to $R^{n}$

$$\log\int_{R^{n}}{\left(2\pi \epsilon^{2}\right)}^{-n/2}\exp\left(-\frac{Y^{T}Q_{\alpha }Y}{2\epsilon^{2}}\right)dY.$$

This integral then simplifies to the log determinant of $Q_{\alpha }^{-1/2}$

$$-\frac{1}{2}\log\left(\det\left(Q_{\alpha }\right)\right),$$

which can also be expressed in terms of the eigenvalues of the design matrix $X^{T}X$, denoted here by $\lambda_{i}$

$$-\frac{1}{2}\log\left(\det\left(Q_{\alpha }\right)\right)=\sum_{i=1}^{m}\log\left(1+\frac{\lambda_{i}}{\alpha }\right).$$

This penalty describes how well we can fit various possible datasets using regression with a ridge constant of α and a gram matrix $X^{T}X$ that has the given spectrum $\left\{\lambda_{1},\lambda_{2},\ldots \;\lambda_{m}\right\}$. Lastly, we need the other component of the stochastic complexity, which is the negative log of the numerator in Equation (7), and also the log likelihood of *Y* under its own parameter fit $-\log P(Y|\theta_{Y,\alpha }^{*};M,X)$. Together, the stochastic complexity becomes the following function of the ridge penalty:

(8) $$L_{\mathrm{SC}}\left(Y;M,X,\alpha \right)=\frac{n}{2\epsilon^{2}}\sigma_{Y,\alpha }^{2}+\sum_{i=1}^{m}\log\left(1+\frac{\lambda_{i}}{\alpha }\right)+\mathrm{const}.$$

The constant term, which we ignore because it is irrelevant for our purpose, is $n\log(\sqrt{2\pi \epsilon })$.

The entries of the m×m gram matrix $X^{T}X$ scale proportionally to *n*, as do its eigenvalues $\lambda_{i}$. This means that in the asymptotic limit n→∞, the complexity penalty in Equation (8) can be written as follows

$$\sum_{i=1}^{m}\log\left(1+\frac{\lambda_{i}}{\alpha }\right)\to \sum_{i=1}^{m}\log\left(\frac{\lambda_{i}}{\alpha }\right)=\sum_{i=1}^{m}\log\left(\lambda_{i}\right)-m\log\left(\alpha \right),$$

Which is equivalent to the Bayesian information penalty

$$\sum_{i=1}^{m}\log\left(1+\frac{\lambda_{i}}{\alpha }\right)\;\mathrm{scales}\;\mathrm{as}\;O\left(1\right)+m\log\left(n\right).$$

This is a standard feature of MDL-derived selection criteria. We also demonstrate in Appendix C that the modified stochastic complexity in Equation (8) is universal in the expected sense [21]. This means that for any data-generating distribution from our parametric model class, the expected efficiency of our NML scheme is optimal in the asymptotic limit n→∞.

Calculating the stochastic complexity requires choosing a variance $\epsilon^{2}$ for the multivariate Gaussian used in our coding scheme. This is also the case for various other ridge penalty criteria [26,27]. A typical method for doing so in the under-parameterized case (m<<n) is to estimate ϵ in terms of the adjusted mean square error of the linear regression solution $\epsilon^{2}=n\sigma_{Y,0}^{2}/\left(n-m\right)$. A more robust method, which is not limited to the under-parameterized case, is provided by Liu et al. [28]. They suggest approximating $\epsilon^{2}$ by the following expression

$$\epsilon^{2}=\frac{Y^{T}\left(I-M_{\alpha^{\prime }}\right)Y}{n-\mathrm{Tr}(M_{\alpha^{\prime }})},$$

which makes use of the projection matrix $M_{\alpha^{\prime }}$. We denote the ridge strength used in this projection matrix by $\alpha^{\prime }$ to distinguish it from the strength α used in model training and the calculation of stochastic complexity.

Our formulation for optimizing the ridge penalty passes two sanity checks that we can construct from our understanding of ridge regression. Firstly, the optimal penalty scales correctly with the terms of the state matrix. If *X* is multiplied by a scalar X→c∗X, the optimal ridge penalty should scale proportionally to its square $\alpha \to c^{2}\alpha$, since this leaves the projection matrix $X{\left(X^{T}X+\alpha I\right)}^{-1}X^{T}$ unchanged. This is the case. The matrix $Q_{\alpha }$ is also invariant to the scaling $\left(X_{ij},\alpha \right)\to \left(cX_{ij},c^{2}\alpha \right)$, which means that the performance term $\frac{n}{2\epsilon }\sigma_{Y,\alpha }^{2}=\frac{1}{2\epsilon }Y^{T}Q_{\alpha }Y$ and the complexity penalty $-\frac{1}{2}\log\left(\det\left(Q_{\alpha }\right)\right)$ are both unaffected. Secondly, since the penalty term is a measure of model flexibility, it should only depend on the unique, non-zero columns in *X*. This is also true. Duplicate columns, or columns of zeros, add eigenvalues of zero to the spectrum and these do not contribute to the penalty term.

## 5. Alternative Approaches 

In Section 3, we accounted for regularization by devising a coding scheme from an ensemble of regularized parameter fits. An alternative approach is to build the ensemble from maximum posterior likelihood estimates after assuming a prior distribution over parameter space. This is similar to the way that luckiness normalized maximum likelihood (LNML) assumes a so-called ‘luckiness function’ $p_{\mathrm{luck}}\left(\theta \right)$ to derive a coding scheme which is optimized for the corresponding luckiness normalized probability density:

(9) $$P_{\mathrm{LNML}}\left(Y;M,X\right):=\frac{\max_{\theta }\left[P\left(Y|\theta ;M,X\right)p_{\mathrm{luck}}\left(\theta \right)\right]}{\int_{Y^{\prime }\in Y}\max_{\theta }\left[P\left(Y^{\prime }|\theta ;M,X\right)p_{\mathrm{luck}}\left(\theta \right)\right]dY^{\prime }}.$$

Dwivedi et al. [20] follow this approach and choose $p_{\mathrm{luck}}$ to be Gaussian

$$p_{\mathrm{luck}}\left(\theta \right)\propto \exp\left(-\frac{\alpha }{2\epsilon^{2}}\theta^{T}\theta \right).$$

This choice is meaningful because a Gaussian prior over the parameters produces a posterior likelihood which is maximal at the ridge regression solution. The numerator and denominator therefore both measure posterior likelihoods. Their resulting luckiness normalized likelihood can be expressed as follows

(10) $$P_{\mathrm{Dwivedi}}\left(Y;M,X\right):=\frac{P\left(Y|\theta_{Y,\alpha }^{*};M,X\right)p_{\mathrm{luck}}\left(\theta_{Y,\alpha }^{*}\right)}{\int_{Y^{\prime }\in Y}P\left(Y^{\prime }|\theta_{Y^{\prime },\alpha }^{*};M,X\right)p_{\mathrm{luck}}\left(\theta_{Y^{\prime },\alpha }^{*}\right)dY^{\prime }}.$$

The description length associated with this probability density, which we denote by $L_{\mathrm{Dwivedi}}$ and provide below, is similar to our own, but includes an additional log parameter likelihood term

$$L_{\mathrm{Dwivedi}}\left(Y;M,X,\alpha \right)=\frac{n}{2\epsilon^{2}}\sigma_{Y,\alpha }^{2}+\frac{\alpha {\left(\theta_{Y,\alpha }^{*}\right)}^{T}\theta_{Y,\alpha }^{*}}{2\epsilon^{2}}+\frac{1}{2}\sum_{i=1}^{m}\log\left(1+\frac{\lambda_{i}}{\alpha }\right).$$

The integral in the denominator also evaluates to half the value of the complexity penalty that we found from the denominator in Equation (7). This can be seen from the way that the prior and posterior probability factorize

$$\exp\left(-\frac{1}{2\epsilon }Y^{T}Q_{\alpha }Y+\alpha {\left(\theta_{Y,\alpha }^{*}\right)}^{T}\theta_{Y,\alpha }^{*}\right)=\exp\left(-\frac{1}{2\epsilon }Y^{T}Q_{\alpha }^{1/2}Y\right).$$

The approach taken by Dwivedi et al. can be generalized to any regularization techniques that offer a Bayesian interpretation. The luckiness function would simply be replaced with whatever prior distribution is implicitly assumed over the parameters.

Another MDL-inspired approach taken by Silhavy et al. [29] makes use of a two-part coding scheme. They take the description length found for linear regression by Giurcaneanu et al. [30] and consider the additional cost of encoding the ridge parameter (or Lasso parameter). It should be noted that Giurcaneanu et al. found the description length for linear regression under a different framing of the model class. They encode the error variance $\sigma_{Y,\alpha =0}^{2}$ and then use the variance to encode the errors $Y∼N(X\theta ,\sigma_{Y,\alpha =0}^{2}I)$. As such, Silhavy et al.’s method for choosing an MDL ridge parameter is quite different than ours. They seek to minimize the following criterion

(11) $$L_{\mathrm{Silhavy}}\left(Y;M,X,\alpha \right):=\frac{n-k}{2}\log\left(\sigma_{Y,\alpha }^{2}\right)+\frac{1}{2}\log\left(\alpha \right)+\frac{k}{2}\log\left({\left(\theta_{Y,\alpha }^{*}\right)}^{T}X^{T}X\theta_{Y,\alpha }^{*}\right)+\mathrm{const}.$$

It should also be noted that the major objective of Silhavy et al.’s work was to apply MDL to multi-criteria decision analysis. This means seeking a tradeoff between model complexity and a combination of various performance measures. In this paper, we only deal with a single objective, which is model log likelihood (effectively the mean square model error).

Other information criteria, like the Akaike [31] and Bayesian [32] Information Criteria (AIC and BIC, respectively), can also be used to choose a ridge strength if the number of model parameters is replaced with the number of effective degrees of freedom, which has dependence on α [33,34]. The commonly used measure of effective degrees of freedom is the trace of the projection matrix $M_{\alpha }=X{\left(X^{T}X+\alpha I\right)}^{-1}X^{T}$ [35,36]. This results in the modified AIC and BIC criteria provided below

$${\mathrm{AIC}}_{\mathrm{eff}}\left(Y;X,\alpha \right)=\frac{n}{\epsilon^{2}}\sigma_{Y,\alpha }^{2}+2\sum_{i=1}^{m}\left(\frac{\lambda_{i}}{\lambda_{i}+\alpha }\right),\;\mathrm{and}$$

$${\mathrm{BIC}}_{\mathrm{eff}}\left(Y;X,\alpha \right)=\frac{n}{\epsilon^{2}}\sigma_{Y,\alpha }^{2}+\log\left(n\right)\sum_{i=1}^{m}\left(\frac{\lambda_{i}}{\lambda_{i}+\alpha }\right).$$

## 6. A Visualization of Probability Density on a Toy Problem 

**Figure 1:**
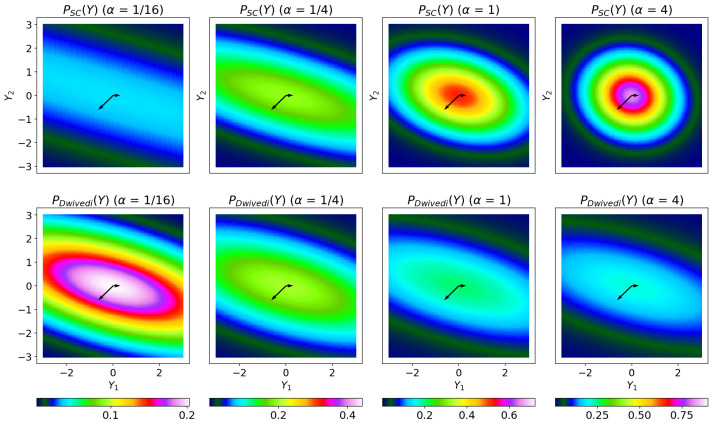
The probability density $P_{\alpha }^{*}\left(Y\right)$ (top row) over the space of possible datasets $Y\in R^{2}$ with varying strengths of ridge regularization (increasing from top left to top right). Density in each column of subplots is indicated by the color scheme beneath. The elliptic structures are level curves of the quadratic form induced by the matrix $Q_{\alpha }$. For comparison, the luckiness normalized density used by Dwivedi et al. is graphed for the same regularization strengths in the bottom row. The elliptic structures in the bottom row are level curves of posterior likelihood, which are induced by the matrix $Q_{\alpha }^{1/2}$ rather than $Q_{\alpha }$. In both cases, probability density is only graphed over a square centered at the origin, but we calculated it by considering the space of datasets to be the entire two-dimensional plane $Y=R^{2}$. The black arrows are position vectors of the rows in *X*.

By modifying regret to account for regularization in Section 3, we alter the NML probability density $P_{\alpha }^{*}$ used to assign description lengths to datasets. This redistributes density towards the datasets whose likelihoods are reduced the least when switching from MLE parameters $P(Y|\theta_{Y}^{*})$ to regularized fits $P(Y|\theta_{Y,\alpha }^{*})$. In Figure 1, we visualize $P_{\alpha }^{*}$ on a toy example. We also visualize the LNML density used by Dwivedi et al. [20] for comparison. The example provided comes from fitting a linear model that maps the following 2×2 input matrix

$$X=\left(\begin{array}{cc} -0.64 & -0.61\\ 0.32 & 0.01 \end{array}\right),$$

to possible output datasets ($Y_{1},Y_{2}$) in $R^{2}$. The values in this example were generated by random sampling of the unit Gaussian and truncated to two decimal places.

When accounting for ridge regularization on linear models, $P_{\alpha }^{*}$ assigns equal probability density to the surfaces of the ellipsoids formed by the matrix $Q_{\alpha }$ (meaning the level sets of the quadratic form $Y^{T}Q_{\alpha }Y=\mathrm{Const}$). These are also surfaces of equal description length in dataset space. As the ridge penalty α is increased, these ellipsoids become smaller and more spherical. The density of $P_{\alpha }^{*}$ is increased close to the origin and decreased for datasets with large entries. However, more generally speaking, the geometry of $P_{\alpha }^{*}$ depends on the model class and the type of regularization performed.

## 7. Fitting a Reservoir Computer 

As a proof of concept, we use our ridge penalty criterion to select a penalty strength for fitting reservoir computer readouts. Reservoir computers are high-dimensional dynamical systems that become conditionally stable when driven by an input signal. Their dynamic response is mapped to the future state of the input with a linear readout. This mapping is possible because the reservoir state-space becomes a time-delay embedding-space of the driving time series [37] according to the echo-state property [38,39], which reservoirs must satisfy. Reservoir readouts are often fitted with ridge regression [40,41,42] and their complexity is a subject of interest [5,43,44,45]. In particular, we use echo-state networks [38] (ESNs) as our reservoirs and perform time series prediction on Lorenz-96 [46]. Our exact method is detailed in Appendix D along with a description of echo-state networks.

**Figure 2:**
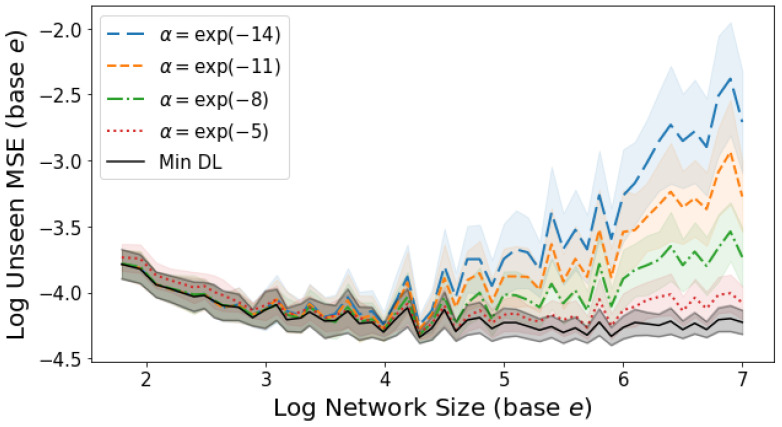
Reservoir performance compared with network size for different choices of regularization strength. The black line represents the performance (as measured by average log mean square testing error) achieved by the ridge strength that minimizes our selection criterion. The colored lines represent the performances achieved by using various fixed ridge penalty strengths. results are averaged over 200 samples and the error bounds indicate twice the standard deviation of bootstrap means.

**Figure 3:**
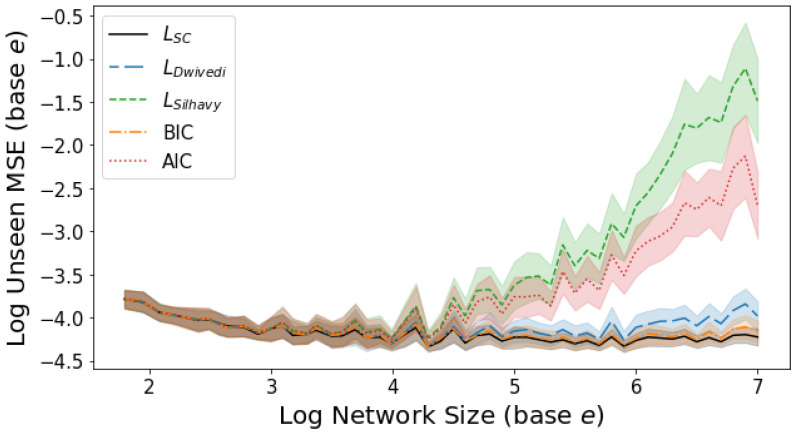
Reservoir performance compared with network size for different choices of regularization strength. The black line represents the performance (as measured by average log mean square testing error) achieved by the ridge strength that minimizes our selection criterion. The colored lines represent the performances achieved using the ridge strengths selected by the other information theoretic criteria discussed in Section 5. Results are averaged over 200 samples and the error bounds indicate twice the standard deviation of bootstrap means.

The average generalization error for one-step prediction of Lorenz-96 is compared with network size in Figure 2. The ridge penalty chosen according to stochastic complexity (in black) produces smaller generalization error on average than any fixed penalty magnitude. This is true across a range of network sizes and suggests that our method leads to sensible choices for the ridge parameter. Our criterion also achieves comparable results to some of the alternative approaches mentioned in Section 5, and performs comparatively well on large networks. This is demonstrated in Figure 3. It should be noted that the relative strength of the model complexity term is dependent on the value of ϵ for these methods, which we choose to be the smaller of the two values described in Section 4

$$\epsilon^{2}=\min\left(\frac{n\sigma_{Y,0}^{2}}{n-k},\frac{Y^{T}\left(I-M_{\alpha^{\prime }}\right)Y}{n-\mathrm{Tr}(M_{\alpha^{\prime }})}\right).$$

The first of these values is always positive and defined, since the length of the training time series is 1200 points and the largest network has 1096 nodes. The latter is calculated using a fixed value of $\alpha^{\prime }=\exp\left(-14\right)$, which we select because the resulting variance estimate $\epsilon^{2}$ tends to be stable for perturbations around this value, and a regularization strength of α=exp(−14) tends to produce well-performing echo-state network readouts.

**Figure 4:**
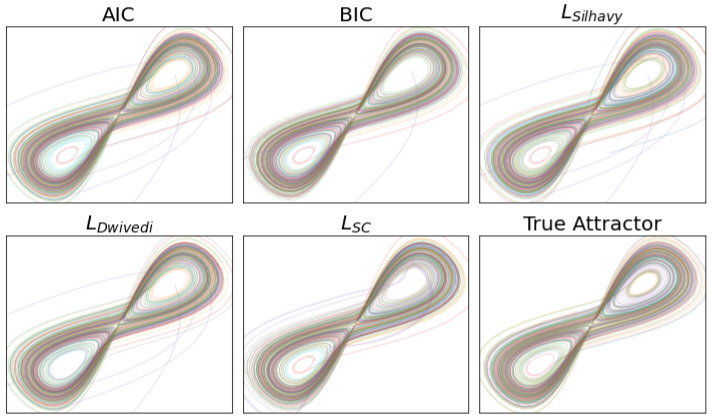
Composite time-delay embedding plots made from 50 trajectories autonomously reconstructed by echo-state networks. Each trajectory (represented in a different color) consists of 200 points predicted by a different network trained on a different integration of Lorenz. The same 50 networks, which each posses 200 nodes, are used to create each subplot. The difference between subplots, except for the bottom right sub-plot, is the ridge strength used to train the ESN readouts. In each case, these strengths were selected by the criterion stated in the title. ESNs were trained to predict the state of the Lorenz attractor 0.02 s into the future. The plot in the bottom right corner contains 50 trajectories numerically integrated from the governing Lorenz equations and represents the set of ideal reconstructions for comparison.

**Figure 5:**
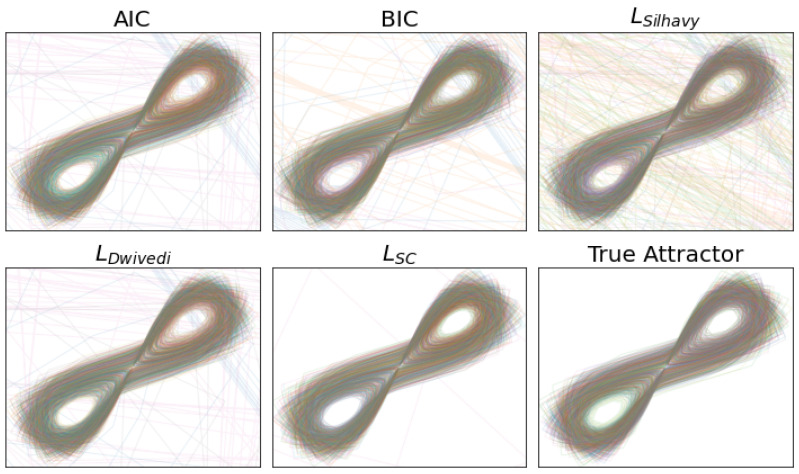
Composite time-delay embedding plots made from 50 trajectories autonomously reconstructed by echo-state networks, like in Figure 4. Each trajectory (represented by a different color) consists of 200 points predicted by a different network trained on a different integration of Lorenz. The same 50 networks are to create each subplot. However, networks are larger than those used in Figure 4, this time containing 800 nodes. The difference between subplots, except for the bottom right, is the ridge strength used to train the ESN readouts. The strengths are selected by the criterion stated in the title. ESNs were trained to predict the state of the Lorenz attractor further into the future (0.1 s) than those in Figure 4. The plot in the bottom right corner contains 50 trajectories numerically integrated from the governing Lorenz equations and represents the set of ideal reconstructions for comparison.

Qualitative comparisons of reconstruction ability are also provided in Figure 4 and Figure 5. This time, echo-state networks are trained for one-step-ahead prediction of the Lorenz system. Individual trajectories in each subplot are generated by continuously driving echo-state networks with their own past predictions. These predictions depend on output coefficients, which in turn depend on the ridge strength selected by each criterion. All criteria tend to produce reconstructions that are qualitatively similar to the Lorenz attractor when networks and prediction step sizes are small. This indicates that the ridge strengths they select tend to be appropriate for capturing the underlying dynamics. For example, the reconstruction composites in Figure 4 are made using 200 node networks trained at a sampling rate of 0.02 s. On the other hand, ridge strengths selected by stochastic complexity appear to produce reasonable reconstructions more often when networks and step sizes are larger. This is the case in Figure 5, where predictions are made using 800 node networks and a step size of 0.1 s.

## 8. Results on PMLB Datasets 

In this section, we replicate the benchmark testing performed by Dwivedi et al. [20] on the publicly available real-world datasets from the PMLB GitHub repository [22] (v1.0.2). Of the 122 datasets in the repository that are intended for regression, we ignore those that have fewer than 5 features, and test the information theoretic criteria discussed in this paper on the remaining 109 datasets. These datasets are sourced from a wide range of research areas.

The original experiments performed by Dwivedi et al. [20] use cross-validation for benchmarking and establish that their method performs well in comparison. This is the case in particular when data are scarce. For the sake of introducing a second point of comparison, we instead perform benchmarking with Stein’s unbiased risk estimate [47,48] (SURE).

**Figure 6:**
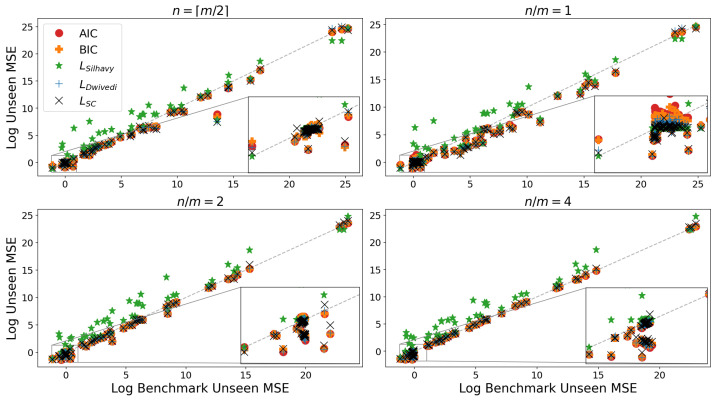
Average log testing MSE (base *e*) using various information theoretic ridge strength selection criteria compared with the average log testing MSE achieved using Stein’s unbiased risk estimate (SURE). Every point in each sub plot represents the average results achieved on a different dataset from the PMLB repository across 50 repetitions of the experiment. On the top left, models are trained using half as many data points *n* as the number of features each dataset contains *m* (with rounding up for datasets with an odd number of features). On the top-right, the ratio of training data points to features is 1, and on the bottom left and right, the ratio is 2 and 4, respectively. Points below the dashed line in each subplot represent datasets on which better average testing performance was achieved by the criterion than by SURE.

In Figure 6, we plot the average testing MSE achieved with each information theoretic criteria against the average achieved using SURE. The results in each subplot are achieved using a different ratio of training data points *n* to the number of dataset features *m* (which is also the number of tunable parameters). These ratios range from over-parameterized (n=⌈m/2⌉) to moderately parameterized (n=4m). In each of the 50 samples that we average across to produce the results in Figure 6, a random subset of rows is selected from each dataset for training, and the rest are reserved for testing. We estimate the variance hyper-parameter $\epsilon^{2}$ using Liu’s method [28] whenever the ratio of *n* to *m* is less than one. Otherwise, like in the previous section, we take the smaller of the estimates provided by the methods discussed in Section 4. However, because the typical scale of data varies widely between datasets in the PMLB repository, we adjust the value of $\alpha^{\prime }$ used in Liu’s method by the average squared magnitude ${\bar{x}}^{2}$ of the entries in each dataset ($\alpha^{\prime }=\exp\left(-14\right){\bar{x}}^{2}$).

In the highly-parameterized settings (1/2≤n/m≤2) the information theoretic criteria discussed in this paper tend to perform well in comparison to the benchmark performance provided by SURE and similarly to one another. This aligns with the findings of Dwivedi et al. [20] and the comparison they drew with cross-validation. As the number of data points used for training is increased beyond the highly-parameterized settings, performances across all methods, including the benchmark, tend to become more similar.

## 9. Summary and Comments 

We have proposed a selection criterion for the choice of penalty strength in ridge regression. In the process, we have demonstrated an application of stochastic complexity to a common problem. This required that we devise a model encoding scheme in accordance with the typical stochastic complexity scheme, but which is sensitive to the application of regularization. Our scheme suggests that we can consider the reduction in model complexity induced by regularization as a constraint on model flexibility. We believe that this result is consistent with the intuitive message espoused by stochastic complexity—that we should be hesitant in choosing a model class that fits our data well if it provides good fits to unrelated datasets using the same predictors. Regularization should ease our hesitancy by preventing our model from easily fitting these unrelated datasets.

The ridge penalty selection criterion that we derived produced promising results when used to fit linear readouts for echo-state networks. It also performed well in highly-parameterized settings on real-world datasets from the PMLB repository when compared to the benchmark provided by Stein’s unbiased risk estimate. However, this performance was well aligned with other information-theoretic criteria.

## Data Availability

The data that support the findings of this study are openly available in the github repository Stochastic_Complexity_for_RR, accessible at https://github.com/antony-mizzi/Stochastic_Complexity_for_RR (accessed on 5 June 2026 ).

## Abbreviations

The following abbreviations are used in this manuscript:

GLM	Generalized Linear Model
MDL	Minimum Description Length
NML	Normalized Maximum Likelihood
LNML	Luckiness Normalized Maximum Likelihood
AIC	Akaike Information Criterion
BIC	Bayesian Information Criterion
ESN	Echo-State Network
MSE	Mean Square Error
SURE	Stein’s Unbiased Risk Estimate

## Appendix A. Demonstration of Minimax Regret Under Proportionality

In the original formulation of stochastic complexity, a coding scheme for possible datasets Y∈Y is optimized according to the probabilities assigned by the distribution $P_{0}^{*}$ that uniquely minimizes worst-case regret as defined in Section 2. It was first proven by Shtarkov [49] that worst-case regret is minimal when $P_{0}^{*}$ is proportional to maximum likelihood $P_{0}^{*}\left(Y\right)\propto P\left(Y|\theta_{Y}^{*}\right)$. The simpler argument that we use in this demonstration is borrowed from Grunwald [21] (Chapter 6.1.2.)

Recall the modified definitions of regret

$$R_{\alpha }\left(Y,P_{\alpha }^{*}\right):=\log\left(P\left(Y|\theta_{Y,\alpha }^{*}\right)\right)-\log\left(P_{\alpha }^{*}\left(Y\right)\right)\equiv -\log\left(\frac{P_{\alpha }^{*}\left(Y\right)}{P(Y|\theta_{Y,\alpha }^{*})}\right),$$

and worst case regret

$$R_{\alpha }^{\max}\left(Y,P_{\alpha }^{*}\right):=\max_{Y\in Y}\left(R_{\alpha }\left(Y,P_{\alpha }^{*}\right)\right),$$

provided in Section 3. We first establish that regret is constant across datasets (meaning $R_{\alpha }\left(Y_{1},P_{\alpha }^{*}\right)=R_{\alpha }\left(Y_{2},P_{\alpha }^{*}\right)\;\forall Y_{1},Y_{2}\in Y$) if and only if $P_{\alpha }^{*}$ is proportional to the likelihood of regularized fits ($P_{\alpha }^{*}\propto P\left(Y|\theta_{Y,\alpha }^{*}\right)$). This is because it follows from our definition of regret that equivalence for different datasets requires equal ratios of their probabilities to the likelihoods of their regularized fits

$$R_{\alpha }\left(Y_{1},P_{\alpha }^{*}\right)=R_{\alpha }\left(Y_{2},P_{\alpha }^{*}\right)\Rightarrow \frac{P_{\alpha }^{*}\left(Y_{1}\right)}{P(Y_{1}|\theta_{Y_{1},\alpha }^{*})}=\frac{P_{\alpha }^{*}\left(Y_{2}\right)}{P(Y_{2}|\theta_{Y_{2},\alpha }^{*})}:=\frac{1}{C_{\alpha }}.$$

For any distribution $P^{\prime }$ defined on Y that is different to $P_{\alpha }^{*}$, there must be some dataset Y∈Y to which it assigns a lesser probability than $P_{\alpha }^{*}$

$$\exists Y\in Y\;s.t.\;P^{\prime }\left(Y\right)<P_{\alpha }^{*}\left(Y\right).$$

If no such dataset exists, then $P^{\prime }$ does not integrate to one and is not a valid distribution. The regret attained by $P^{\prime }$ on this dataset must be greater than the worst case regret attained by $P_{\alpha }^{*}$

$$\log\left(P\left(Y|\theta_{Y,\alpha }^{*}\right)\right)-\log\left(P^{\prime }\left(Y\right)\right)>\log\left(P\left(Y|\theta_{Y,\alpha }^{*}\right)\right)-\log\left(P_{\alpha }^{*}\left(Y\right)\right)=\log\left(C_{\alpha }\right).$$

It follows that the worst case regret under $P^{\prime }$ is greater than under $P_{\alpha }^{*}$. This is true for any arbitrary distribution $P^{\prime }$ that is different to $P_{\alpha }^{*}$, so $P_{\alpha }^{*}$ is the unique distribution that achieves minimax regret.

## Appendix B. Conditions for a Well-Defined Coding Scheme

As discussed in Section 3 and illustrated in Section 6, by adjusting the notion of regret to account for regularization, we change the probability density of the resulting NML distribution $P_{\alpha }^{*}$. The coding scheme produced by this new distribution will remain well-defined in all contexts where the scheme produced without regularization is well-defined, so long as it stays non-zero over the original support $P_{\alpha }^{*}\left(Y\right)>0\;\forall Y\in Y$. This is the case if the following two requirements are satisfied. Firstly, the normalization factor must not be made zero or infinite by regularization,

$$\begin{array}{cc} 0 & <\int_{Y^{\prime }\in Y}P\left(Y^{\prime }|\theta_{Y^{\prime },\alpha }^{*};M,X\right)dY^{\prime }<\infty \;\mathrm{in}\;\mathrm{all}\;\mathrm{cases}\;\mathrm{where}\;\\ 0 & <\int_{Y^{\prime }\in Y}P\left(Y^{\prime }|\theta_{Y^{\prime }}^{*};M,X\right)dY^{\prime }<\infty . \end{array}$$

Secondly, model likelihoods at regularized parameter fits must remain non-zero over the full support of $P_{0}^{*}$, which we can write as follows,

$$\theta_{Y,\alpha }^{*}\;\mathrm{exists},\;\mathrm{and}\;P\left(Y|\theta_{Y,\alpha }^{*}\right)>0\;\forall Y\in Y.$$

Together, these requirements ensure that codeword lengths defined by our modified form of stochastic complexity

$$L_{\mathrm{SC}}\left(Y;M,X,\alpha \right)=-\log\frac{P(Y|\theta_{Y,\alpha }^{*};M,X)}{\int_{Y^{\prime }\in Y}P\left(Y^{\prime }|\theta_{Y^{\prime },\alpha }^{*};M,X\right)dY^{\prime }}$$

are finite for every possible dataset, even when the space of possibilities is the full support of $P_{0}^{*}$.

The first of the two requirements is always satisfied. Probability is greatest at the maximum likelihood estimate $P\left(Y|\theta_{Y}^{*}\right)\geq P\left(Y|\theta_{Y,\alpha }^{*}\right)$, which means that the modified normalization factor (Equation (6)) is never more than the original normalization factor (Equation (4)). It also does not make sense for the normalization factor to be reduced to zero. On the contrary, there are combinations of model classes and regularization techniques, like linear models and ridge regression, that together produce a finite normalization factor despite the normalization factor being infinite without regularization.

The second requirement is satisfied for most conceivable model classes and types of regularization. For model likelihoods to be reduced to zero by regularization, the distribution used to assign model likelihood must have restricted support. We used a multivariate Gaussian to assign likelihoods to linear models, which means that likelihood is positive no matter how poor the regularized fit (P(Y|θ)>0∀Y,θ). Even if the distribution used to assign likelihood has restricted support, regularization still needs to make it possible to assign a best-fit likelihood of zero. For example, no strength of L2 or L1 regularization could produce zero likelihood fits ($P(Y|\theta_{Y,\alpha }^{*})=0$) in any scenario where maximum likelihood is non-zero ($P(Y|\theta_{Y}^{*})>0$). This is because they both maximize posterior likelihood after assuming priors π(θ) that have universal support. Assigning a zero-likelihood regularized fit when a non-zero maximum likelihood exists would require the following impossibility

$$\max_{\theta \in \Theta }\left(P(Y|\theta )\pi (\theta )\right)=0\;\mathrm{despite}\;P\left(Y|\theta_{Y}^{*}\right),\;\pi \left(\theta_{Y}^{*}\right)>0\;\mathrm{for}\;\mathrm{some}\;\theta_{Y}^{*}\in \Theta .$$

In classification tasks, models typically assign probabilities to each category in such a way that probabilities are strictly greater than zero and less than one. This makes it impossible for regularized parameter fits to ever assign dataset likelihoods of zero and violate the second requirement. Stochastic complexity is also reduced to its discrete form in categorization tasks, which means that the normalization factor is always bounded above by the finite number of possible datasets, and the first requirement is always satisfied.

## Appendix C. Universality in Expectation

For the NML distribution derived in Section 4 to constitute a universal model in the expected sense, its efficiency must converge on the optimal efficiency for any possible data-generating process as the number of data points tends to infinity [21]. The data-generating processes in consideration are those that can be generated by any parameterization of the model class used to derive the coding scheme. In our case, these are the multivariate normal distributions with variance $\epsilon^{2}$ and mean Xθ. The requirement for universality is satisfied if expected regret ($R_{0}$) per symbol tends to zero in the asymptotic limit for dataset size. Expectation is taken with respect to the probability of each dataset $y^{n}$ given the data generating process. Here we denote datasets by $y^{n}$ to indicate their length. We also denote data-generating distributions by $P_{\theta }$, under which the probability of each dataset is conditional on some true parameter vector $P_{\theta }\left(y^{n}\right)=P\left(y^{n}|\theta \right)$. As such, we can write the requirement for universality in the expected sense using the definition of $R_{0}$ from Section 2

$$\begin{array}{cc} \frac{1}{n}E_{P_{\theta }}\left[R_{0}\left(y^{n},P_{\alpha }^{*}\right)\right] & :=\frac{1}{n}\int R_{0}\left(y^{n},P_{\alpha }^{*}\right)P\left(y^{n}|\theta \right)dy^{n}\to 0\;\mathrm{as}\;n\to \infty \\ \mathrm{where}\;R_{0}\left(y^{n},P_{\alpha }^{*}\right) & =\log(P\left(y^{n}|\theta_{y^{n}}^{*})\right)-\log\left(P_{\alpha }^{*}\left(y^{n}\right)\right)=\log\frac{P(y^{n}|\theta_{y^{n}}^{*})}{P_{\alpha }^{*}\left(y^{n}\right)}. \end{array}$$

We can also re-write regret using the expression derived for $P_{\alpha }^{*}$ in Section 4

$$R_{0}\left(y^{n},P_{\alpha }^{*}\right)=\log\frac{\exp(-\frac{1}{2\epsilon^{2}}{\left(y^{n}\right)}^{T}Q_{0}y^{n})}{\exp(-\frac{1}{2\epsilon^{2}}{\left(y^{n}\right)}^{T}Q_{\alpha }y^{n})}+\sum_{i=1}^{m}\log\left(1+\frac{\lambda_{i}}{\alpha }\right),$$

and further simplify it to the following

(A1) $$R_{0}\left(y^{n},P_{\alpha }^{*}\right)=\frac{1}{2\epsilon^{2}}{\left(y^{n}\right)}^{T}\left(Q_{\alpha }-Q_{0}\right)y^{n}+\sum_{i=1}^{m}\log\left(1+\frac{\lambda_{i}}{\alpha }\right).$$

The complexity penalty $\sum_{i=1}^{m}\log\left(1+\frac{\lambda_{i}}{\alpha }\right)$ has no dependence on the dataset $y^{n}$. As such, we can exclude it from the expectation and re-write our requirement

(A2) $$\frac{1}{n}E_{P_{\theta }}\left[\frac{1}{2\epsilon^{2}}{\left(y^{n}\right)}^{T}\left(Q_{\alpha }-Q_{0}\right)y^{n}\right]+\frac{1}{n}\log\left(1+\frac{\lambda_{i}}{\alpha }\right)\to 0\;\mathrm{as}\;n\to \infty .$$

As discussed in Section 4, the complexity penalty is asymptotically equivalent to the BIC penalty and scales as log(n). This means that the second term in Equation (A2) vanishes as *n* is taken to infinity at a rate of log(n)/n. We are left with only the requirement that the first term in Equation (A2) vanishes. Given that $P_{\theta }$ is the multivariate normal density function, we can evaluate the expectation value and write this requirement as follows

(A3) $$\frac{1}{n}\left(\frac{{\left(\mu^{n}\right)}^{T}\left(Q_{\alpha }-Q_{0}\right)\mu^{n}}{2\epsilon^{2}}+\frac{1}{4\epsilon^{2}}\mathrm{Tr}\left(Q_{\alpha }-Q_{0}\right)\right)\to 0,$$

where $\mu^{n}$ is the expected dataset given the generating distribution ($\mu^{n}=X\theta$).

To show that Equation (A3) holds in the asymptotic limit for *n*, we can factorize the matrix $Q_{\alpha }-Q_{0}$ using the definition of $Q_{\alpha }$ provided in Section 4

$$Q_{\alpha }-Q_{0}=\left(M_{\alpha }-M_{0}\right)\left(M_{\alpha }+M_{0}-2I\right),$$

and then use the matrix identity $A^{-1}-{\left(A+\alpha I\right)}^{-1}=\alpha A^{-1}{\left(A+\alpha I\right)}^{-1}$

$$Q_{\alpha }-Q_{0}=\alpha X{\left(X^{T}X\right)}^{-1}{\left(X^{T}X+\alpha I\right)}^{-1}X^{T}\left(M_{\alpha }+M_{0}-2I\right).$$

The entries of the m×m gram matrix $X^{T}X$ grow on the order *n*. This means that the entries of the n×n matrix $M_{\alpha }-M_{0}$ that we expanded grow on the order of $1/n^{2}$. Meanwhile, The diagonal entries of $M_{\alpha }+M_{0}$ are bounded between 0 and 2, and the off-diagonal entries are bounded between −2 and 2. This means that the magnitude of all entries in the matrix $M_{\alpha }+M_{0}-2I$ are bounded above by 2. Since the entries of the matrix $Q_{\alpha }-Q_{0}$ are obtained from the product of these matrices

$${\left(Q_{\alpha }-Q_{0}\right)}_{ij}=\sum_{k=0}^{n}{\left(M_{\alpha }-M_{0}\right)}_{ik}{\left(M_{\alpha }+M_{0}-2I\right)}_{kj},$$

they adhere to an upper bound

$$|{\left(Q_{\alpha }-Q_{0}\right)}_{ij}|\leq 2\sum_{k=0}^{n}\left|{\left(M_{\alpha }-M_{0}\right)}_{ik}\right|,$$

which grows on the order of 1/n. This means that the quadratic form ${\left(\mu^{n}\right)}^{T}\left(Q_{\alpha }-Q_{0}\right)\mu^{n}$ and the trace $\mathrm{Tr}(Q_{\alpha }-Q_{0})$, which require an additional summation of *n* terms, are scale invariant. It follows that the expectation value in Equation (A2) also vanishes in the asymptotic limit and $P_{\alpha }^{*}$ achieves universality in the expected sense. This remains true even if the regularization strength α is allowed to grow in conjunction with the number of data-points, so long as the growth rate is sub-linear.

## Appendix D. Echo-State Networks and Time-Series Prediction

Echo-state networks are a specific type of reservoir. They consist of a network and a set of input weights and behave like the recurrent part of a recurrent neural network. At each time step, the network is driven according to the value of the time series u(t). The network state x(t) updates according to the governing equation

$$x\left(t+1\right)=f(Ax\left(t\right)+W^{in}u\left(t\right)+b).$$

Unlike recurrent neural networks, the adjacency matrix *A*, input connections $W^{in}$ and node biases *b* all remain untrained. The activation function *f*, which in our case is the hyperbolic tangent f(·)=tanh(·), introduces non-linearity. Predictions at each time step are given by the linear combination of node values $\theta^{T}x\left(t\right)$ with parameters θ determined from ridge regression $\theta_{Y,\alpha }^{*}={\left(X^{T}X+\alpha I\right)}^{-1}X^{T}Y$. We train echo-state networks for one-step-ahead prediction. In this case, *X* is the matrix with a *t*th row of x(t) and *Y* is the vector with a *t*th entry of u(t+1).

In the experiments performed in Section 7, every sample involves a new ESN architecture and a newly generated time series. We generate the network adjacency *A* as a weighted Watts-Strogatz network with a connectivity of 6 weightings drawn from the standard normal. The adjacency matrix is then normalized to have a spectral radius of ρ, which is sampled uniformly between 3/4 and 3/2. Input connection strengths are sampled from a Gaussian with zero mean and a standard deviation of 0.3. We do not use node biases ($b=\vec{0}$).

For Figure 2 and Figure 3 the driving time series consists of 1300 points generated from a Runge-Kutta (RK4) integration of Lorenz-96 with a sampling rate of 0.1 s and an integration step length of 0.01 s. However, ESNs are only trained according to the final 1200 of these time series points. The first 99 are disregarded to establish conditional dependence of the state of the network on the time series and predictions are trained 1 step ahead. Lorenz96 is a high-dimensional dynamical system with a Lyapunov time that we calculate to be about 0.68 s using the method in [50]. It has a governing set of equations detailed below in Equation (A4), and we use the hyperparameters (R,n)=(8,15).

(A4) $${\dot{x}}_{i}=\left(x_{i+1}-x_{i-2}\right)x_{i-1}-x_{i}+R,$$

Note that in this equation, $x_{0}$ is the same as $x_{n}$.

For the time delay embedding plots (Figure 4 and Figure 5), we instead train the reservoir at one-step prediction using 1200 points from the Lorenz system integrated using an RK4 scheme with a step length of 0.01 s. The driving time series again contains 1300 points but the first 99 are disregarded when training the readout. The governing equations of this system are provided below. We use standard hyperparameters $\left(\sigma ,\rho ,\beta \right)=\left(10,28,\frac{8}{3}\right)$ for which we calculate the Lyapunov time to be around 1.1 s.

$$\begin{array}{cc} \dot{x} & =\sigma (y-x)\\ \dot{y} & =x(\rho -z-y)\\ \dot{z} & =xy-\beta z \end{array}$$

The trajectories shown in Figure 4 and Figure 5 are generated autonomously. This means that rather than driving ESNs by an external signal, we instead drive them at each time step by their previous prediction. Effectively, their predictions are an Eulerian integration of the following system of equations

$$\begin{array}{cc} \hat{u}\left(t\right) & =\theta^{T}x\left(t\right),\\ x(t+1) & =f(Ax\left(t\right)+W^{in}\hat{u}\left(t\right)+b). \end{array}$$

This autonomous prediction is continued for a fixed duration of 200 time steps. For each predicted trajectory $\hat{u}$, We graph $\hat{u}\left(t\right)$ against $\hat{u}\left(t+5\right)$ in Figure 4 and $\hat{u}\left(t\right)$ against $\hat{u}\left(t+1\right)$ in Figure 5. Both plots are comprised of 50 different trajectories, each of which is produced by a different ESN architecture trained on a different time series. The same 50 architectures and time series are used in each subplot.
